# Supplementary material for: Superradiant lasing in inhomogeneously broadened ensembles with spatially varying coupling
Source: Open Res Eur. 2021 Sep 22;1:73. Originally published 2021 Jun 25. [Version 2] doi: 10.12688/openreseurope.13781.2 (PMC10446144; doi:10.12688/openreseurope.13781.2)
Supplement: Peer review history

# Peer review: Reviewer response for version 2

**DOI:** 10.21956/openreseurope.15259.r27658

**Published:** 2021-10-04

**Authors:** Kamanasish Debnath

**Affiliations:** 1 Wallenberg Centre for Quantum Technology, Chalmers University of Technology, Gothenburg, Sweden

The authors have addressed all my comments and I would be happy to approve the article.

Is the study design appropriate and does the work have academic merit?

Yes

Is the work clearly and accurately presented and does it cite the current literature?

Yes

If applicable, is the statistical analysis and its interpretation appropriate?

Not applicable

Are all the source data underlying the results available to ensure full reproducibility?

Yes

Are the conclusions drawn adequately supported by the results?

Yes

Are sufficient details of methods and analysis provided to allow replication by others?

Yes

Reviewer Expertise:

Quantum optics

I confirm that I have read this submission and believe that I have an appropriate level of expertise to confirm that it is of an acceptable scientific standard.

# Peer review: Reviewer response for version 2

**DOI:** 10.21956/openreseurope.15259.r27659

**Published:** 2021-09-27

**Authors:** David Petrosyan

**Affiliations:** 1 Institute of Electronic Structure & Laser, Foundation for Research and Technology-Hellas, GR-70013 Heraklion, Crete, Greece

The authors addressed all the comments and questions clearly and convincingly. I have no further comments on this manuscript.

Is the study design appropriate and does the work have academic merit?

Yes

Is the work clearly and accurately presented and does it cite the current literature?

Yes

If applicable, is the statistical analysis and its interpretation appropriate?

Yes

Are all the source data underlying the results available to ensure full reproducibility?

Yes

Are the conclusions drawn adequately supported by the results?

Yes

Are sufficient details of methods and analysis provided to allow replication by others?

Yes

Reviewer Expertise:

Quantum optics

I confirm that I have read this submission and believe that I have an appropriate level of expertise to confirm that it is of an acceptable scientific standard.

# Peer review: Reviewer response for version 1

**DOI:** 10.21956/openreseurope.14860.r27159

**Published:** 2021-07-07

**Authors:** David Petrosyan

**Affiliations:** 1 Institute of Electronic Structure & Laser, Foundation for Research and Technology-Hellas, GR-70013 Heraklion, Crete, Greece

The authors present the results of their numerical studies of an ensemble of atoms coupled to a common mode of an optical cavity. The atoms are modeled as two-level systems incoherently pumped to the excited state from where they can spontaneously decay to the ground state or coherently emit a photon into the cavity mode. To treat large number of atoms N, the authors uses the method of cumulant expansion up to second order in the field and atomic operators, which permits calculations of the first-order field correlation function, with its Fourier transform being the power spectrum of the emitted radiation.

 It has been shown before that even when the cavity linewidth is much larger than the natural linewidth of the atomic transition, the collective atomic coherence can result in superradiant lasing with very narrow linewidth, many orders of magnitude smaller than that of an empty cavity. This can have many interesting and important applications in spectroscopy, precision measurement, optical clocks, etc. The authors first reproduce these results, and determine the appropriate values of the pumping rate R, the atom number N, and the atom-cavity coupling strength g (also taking into account the cavity dephasing rate ξ), corresponding to the collective strong coupling regime.  

     

 Then the authors study the superradiant lasing from inhomogeneous atomic ensembles, with different atoms having different transition frequencies, but still the same transition linewidth. For moderate pumping, each subensemble of atoms produces superradiant lasing with its own frequency and the total lasing linewidth is that of the inhomogeneous atomic linewidth. But for stronger pumping, a dynamical transition to narrow-line synchronized lasing can occur. The authors also consider spatial variations of the coupling strengths of the atoms to the cavity mode, and show that the superradiant lasing can still occur with the linewidth determined by an effective averages coupling strength g _eff_. Finally, the authors show that detrimental effects of the cavity dephasing can be significantly reduced by introducing additional atomic dephasing. This is a counterintuitive result and it would be useful if the authors can provide a physical interpretation for it.      

 Overall, this is a well-written manuscript presenting a good quality work, which I approve with minor recommendations:

- On page 6, in the third paragraph, it should be the Gaussian distribution of the atomic transition frequencies σ=κ (not atom number).
- In Fig. 4 perhaps it should be stated that what is shown is the normalized intensity (or max intensity is set to 1).
- In Fig. 5, the critical value of the pumping rate vs inhomogeneous linewidth σ is shown for N=10 ^4^ atoms. How does it change with the atom number? Is it approximately inversely proportional to N, for otherwise the same parameters?
- The authors mention that inhomogeneous broadening of the atomic transition can originate from trapping within the cavity, motion, or optical pumping. It would be useful if the author could expand the discussion a bit and clarify these issues. Do they mean that, depending on the atom position in the trap, its transition frequency will be different? Or that atom motion leads to the Doppler frequency shift? How does optical pumping lead to different frequency shifts of different atoms?
- The present manuscript did not explicitly consider high-density atomic ensembles, where the atomic collisions or dipole-dipole interactions would lead to increased coherence relaxation or collective broadening of the atomic transition. Would the authors be in a position to give some informed speculations as to whether the superradiant lasing with narrow line spectrum will still be possible in such situations, compensating thereby the detrimental broadening due to the interatomic interactions?

Is the study design appropriate and does the work have academic merit?

Yes

Is the work clearly and accurately presented and does it cite the current literature?

Yes

If applicable, is the statistical analysis and its interpretation appropriate?

Yes

Are all the source data underlying the results available to ensure full reproducibility?

Yes

Are the conclusions drawn adequately supported by the results?

Yes

Are sufficient details of methods and analysis provided to allow replication by others?

Yes

Reviewer Expertise:

Quantum optics

I confirm that I have read this submission and believe that I have an appropriate level of expertise to confirm that it is of an acceptable scientific standard.

# Response

**Published:** 2021-09-22

**Authors:** Anna Bychek

**Affiliations:** University of Innsbruck, Austria

Dear Dr. Petrosyan, First, we would like to thank you for a thorough reading of the manuscript and your constructive and helpful comments. Below, we respond to your comments point by point (our answer is marked using a different font).  

1. *... the authors show that detrimental effects of the cavity dephasing can be significantly reduced by introducing additional atomic dephasing. This is a counterintuitive result and it would be useful if the authors can provide a physical interpretation for it.* It is well established that incoherent pumping is always tied to atomic dephasing.
  It reduces the coherence stored in the atoms but at the same time broadens the individual atomic emission line and the spectral gain profile. In this way the effective coupling between atoms with different detunings is enhanced. As can also be seen from Eq. 8, pumping and atomic dephasing appear at the same place in the diagonal terms of the matrix *A* and induce similar physical effects. Thus, individual atomic dephasing spreads the atomic gain over a larger frequency range and thus can induce extra atom-atom coupling by enlarging the overlap of distinct spectral lines, which finally leads to better synchronization. We added a corresponding sentence in our paper.
2. *On page 6, in the third paragraph, it should be the Gaussian distribution of the atomic transition frequencies σ=κ (not atom number).*
  Here the Gaussian distribution is related to Figure 4(a) (Figure 3(a) in the original version), where we consider only five frequency clusters *_m_=[- ; - /2; 0; /2; ]*. On this simple example we would like to introduce our concept to the readers, which we use throughout the whole paper. We model the continuous frequency distribution with the standard deviation *σ* by choosing equidistant cluster frequencies, with the number of atoms per cluster given by the Gaussian distribution with the standard deviation *σ*. We have slightly changed the text in order to make it straightforward.
3. *In Fig. 4 perhaps it should be stated that what is shown is the normalized intensity (or max intensity is set to 1).*
  The comment on this has been added to the figure caption.
4. *In Fig. 5, the critical value of the pumping rate vs inhomogeneous linewidth σ is shown for N=10 ^4^ atoms. How does it change with the atom number? Is it approximately inversely proportional to N, for otherwise the same parameters?*
  This result appears to be independent of *N*. For an arbitrary given number of *N* the structure of the spectrum remains the same as in Fig. 5 (Fig. 4 in the original version). In a weak pumping regime, the number of atoms determines the linewidth of each individual line, while after merging it determines the linewidth of a single line. The critical value of pumping which distinguishes the discrete spectrum of clusters from a single line remains the same for any *N*. In the revised version, we give a comparison of the results for *N=10 ^2^* (red dots) and *N=10 ^4^* (blue circles) atoms in Fig.6, which confirms our predictions.
5. *The authors mention that inhomogeneous broadening of the atomic transition can originate from trapping within the cavity, motion, or optical pumping. It would be useful if the author could expand the discussion a bit and clarify these issues. Do they mean that, depending on the atom position in the trap, its transition frequency will be different? Or that atom motion leads to the Doppler frequency shift? How does optical pumping lead to different frequency shifts of different atoms?*
  Here we mainly think of differential Stark shifts e.g. induced by an inhomogeneous trapping lattice or a pump laser with with a Gaussian profile. Actually several lasers are needed to implement an effective pump rate as we use here. We are currently quantitatively studying this effect in a separate effort, which we hope will be finished soon. Doppler shifts would have similar effects in ring cavities, whereas in a standing-wave cavity they would generate a time-dependent atom-field coupling which we do not consider here.
6. *The present manuscript did not explicitly consider high-density atomic ensembles, where the atomic collisions or dipole-dipole interactions would lead to increased coherence relaxation or collective broadening of the atomic transition. Would the authors be in a position to give some informed speculations as to whether the superradiant lasing with narrow line spectrum will still be possible in such situations, compensating thereby the detrimental broadening due to the interatomic interactions?*
  We are considering atoms at fixed positions here, so that direct collisions are left out. The effect of dipole-dipole interactions have been studied in a small scale, full quantum model before (T Maier *et al.* 2014 Optics express 22 (11), 13269-13279) and appear not too detrimental. Similarly, collisions could have even a positive effect for synchronization, but a quantitative guess is hard (B Zhu *et al*. 2015 New J. Phys. 17 083063). We added those references and a comment.

# Peer review: Reviewer response for version 1

**DOI:** 10.21956/openreseurope.14860.r27157

**Published:** 2021-07-07

**Authors:** Kamanasish Debnath

**Affiliations:** 1 Wallenberg Centre for Quantum Technology, Chalmers University of Technology, Gothenburg, Sweden

In the paper “ *Superradiant lasing in inhomogeneously broadened ensembles with spatially varying coupling*”, the authors numerically study superradiant lasing from an incoherently pumped two-level atomic ensemble coupled to a bad cavity. The authors employ second-order mean-field theory and investigate the effect of various noise sources, inhomogeneous broadening, and variable coupling strengths. After a brief introduction and description of the model, the authors describe the second-order cumulant expansion approach which is followed by the results for a single atomic ensemble in the absence of inhomogeneous broadening but with non-zero cavity and atomic dephasing. Finally, the authors introduce inhomogeneous broadening and variable light-matter coupling in their system and study the emission properties.

 I think the results are interesting and is of interest to both theoretical and experimental groups working on superradiant lasing using ultra-cold atoms. In addition, a detailed study of various noise sources and inhomogeneous light-matter coupling as considered in this manuscript was not taken into account in Ref. [11] and [31], so the manuscript definitely fills that gap. I have some minor comments which I believe will improve the clarity of the manuscript. 

1. Since the manuscript emphasizes on various noise sources, I think it would be helpful to include a color plot for the linewidth of the emission where cavity and atomic dephasing is varied along x and y axis with other parameters remaining the same in Fig.2.  This would highlight the tolerance of the linewidth on the two noise sources.
  In addition, the two paragraphs above Fig. 2 where the authors describe the figures was a bit confusing to me, mostly because all the figures were not labelled (a)-(h) and all figures were not described. For example, the authors write- “In Figure 2(c), we scan the linewidth over the coupling strength g ….”, which is actually the lower panel of Fig. 2(c). So I would recommend that a proper labelling of all the figures would make it easy for the readers.
2. Next, the authors introduce inhomogeneous broadening and show that the atoms synchronize beyond a critical pump strength and for a weak pumping the spectrum reveals a single broad peak for sufficiently large M. It would be interesting to know the cross correlations between atoms in different clusters (let’s say between cluster number 1 and 201) as a function of the pump strength. The correlations should be easily available from the second moment equations. I would expect the cross correlations to grow in the synchronized regime, but what about in the regime where the emission reveals a single broad spectrum. Can the authors comment on this?
3. In Fig.4(c), the authors take into account overall atom-number fluctuations. Did the authors introduce another equation for the atom number like dN/dt which models the loss of atoms from the trap? It might be helpful for the readers to add a few lines about how this was done numerically.
4. Was the equation R _c_= 0.4σ derived by fitting the data points? The authors may add a comment on that for clarity. The equation is independent of M, but what about N? Can the authors make an intuitive comment about it?
5. The authors model the inhomogeneous broadening and varying light-matter coupling with M=11 and K=5 clusters. Does this mean it is a 2D grid of 11 x 5 dimensions?

Few typographical errors-

1. ħ in Eq.1 is no longer present in any of the later equations.
2. A minus sign is missing in Eq.4
3. In Eq.8, the correlation terms with superscript “st” is not defined in the text.
4. On page 6, first paragraph last line. I guess the authors meant gain “medium”.

Is the study design appropriate and does the work have academic merit?

Yes

Is the work clearly and accurately presented and does it cite the current literature?

Yes

If applicable, is the statistical analysis and its interpretation appropriate?

Not applicable

Are all the source data underlying the results available to ensure full reproducibility?

Yes

Are the conclusions drawn adequately supported by the results?

Yes

Are sufficient details of methods and analysis provided to allow replication by others?

Yes

Reviewer Expertise:

Quantum optics

I confirm that I have read this submission and believe that I have an appropriate level of expertise to confirm that it is of an acceptable scientific standard, however I have significant reservations, as outlined above.

# Response

**Published:** 2021-09-22

**Authors:** Anna Bychek

**Affiliations:** University of Innsbruck, Austria

Dear Dr. Debnath, First, we would like to thank you for a thorough reading of the manuscript and your constructive comments, which helped us to improve our work. Below, we respond to your comments point by point by using a different font.  

*Since the manuscript emphasizes on various noise sources, I think it would be helpful to include a color plot for the linewidth of the emission where cavity and atomic dephasing is varied along x and y axis with other parameters remaining the same in Fig 2. This would highlight the tolerance of the linewidth on the two noise sources.*
  Thank you for the valuable suggestion - in the revised version we have included an additional figure (Fig. 3) illustrating the influence of both cavity and atomic dephasing on the linewidth.
*In addition, the two paragraphs above Fig. 2 where the authors describe the figures was a bit confusing to me, mostly because all the figures were not labelled (a)-(h) and all figures were not described. For example, the authors write- “In Figure 2(c), we scan the linewidth over the coupling strength g ….”, which is actually the lower panel of Fig. 2(c). So I would recommend that a proper labelling of all the figures would make it easy for the readers.*
  We agree that the labels in Fig. 2 could be misleading. In the revised version, all plots in Fig. 2 are labeled (a)-(h). *2.*
*Next, the authors introduce inhomogeneous broadening and show that the atoms synchronize beyond a critical pump strength and for a weak pumping the spectrum reveals a single broad peak for sufficiently large M. It would be interesting to know the cross correlations between atoms in different clusters (let’s say between cluster number 1 and 201) as a function of the pump strength. The correlations should be easily available from the second moment equations. I would expect the cross correlations to grow in the synchronized regime, but what about in the regime where the emission reveals a single broad spectrum. Can the authors comment on this?*
  This is indeed a question which was interesting to us as well. In the revised version, we decided to include an appendix regarding the cross-correlations between atoms in different clusters. We considered the cross-correlations between the first and the central clusters in Figure 5(a) (Figure 4 in the original version) as a function of the pumping strength. In a weak pumping regime, the correlations are zero and grow with the pumping strength, as shown in Figure 9 in the appendix. The function reaches its maximal value when the ensemble is fully synchronized. However, as pumping continues to grow the correlations decrease due-to growing dephasing imposed by pumping.
*In Fig.4(c), the authors take into account overall atom-number fluctuations. Did the authors introduce another equation for the atom number like dN/dt which models the loss of atoms from the trap? It might be helpful for the readers to add a few lines about how this was done numerically.*
  The total number of atoms always remains constant and equal to N, so there is no loss of atoms form the trap. The particle number fluctuations simply were added in the cluster's atom numbers as small random deviations from a Gaussian distribution in a way that keeps the total number of atoms constant. The comment on this has been added in the text.
*Was the equation R _c_=0.4σ derived by fitting the data points? The authors may add a comment on that for clarity. The equation is independent of M, but what about N? Can the authors make an intuitive comment about it?* Yes, the function *R _c_=0.4σ* was found as the best linear fit to the data points.
  The comment on this has been added to the figure caption. This result appears to be independent of *N*. For an arbitrary given number of *N* the structure of the spectrum remains the same as in Fig. 5 (Fig. 4 in the original version). In a weak pumping regime, the number of atoms determines the linewidth of each individual line, while after merging it determines the linewidth of a single line. The critical value of pumping which distinguishes the discrete spectrum of clusters from a single line remains the same for any *N*. In the revised version, we give a comparison of the results for *N*=10 ^2^ (red dots) and *N*=10 ^4^ (blue circles) atoms in Fig.6, which confirms our predictions.
*5. The authors model the inhomogeneous broadening and varying light-matter coupling with M=11 and K=5 clusters. Does this mean it is a 2D grid of 11 x 5 dimensions?* This is correct. The overall number of clusters in the model is equal to *MxK* clusters of atoms.
*Few typographical errors-*

- *ħ in Eq.1 is no longer present in any of the later equations.*
- *A minus sign is missing in Eq.4*
- *In Eq.8, the correlation terms with superscript “st” is not defined in the text.*
- *On page 6, first paragraph last line. I guess the authors meant gain “medium”.*

Thank you for the helpful remarks. We agree and corrected the errors, except Eq. 4 which is correct, for a reference see, for example, Eq.(94) in Jacobs K, Steck DA. A straightforward introduction to continuous quantum measurement. Contemporary Physics 47(5):279-303 (2006).
